# Adherence to Hemodialysis and Associated Factors among End Stage Renal Disease Patients at Selected Nephrology Units in Rwanda: A Descriptive Cross-Sectional Study

**DOI:** 10.1155/2018/4372716

**Published:** 2018-06-03

**Authors:** Marie Claire Mukakarangwa, Geldine Chironda, Busisiwe Bhengu, Godfrey Katende

**Affiliations:** ^1^School of Nursing and Midwifery, College of Medicine and Health Sciences, University of Rwanda, Kigali, Rwanda; ^2^Human Resources for Health (HRH), University of Rwanda, College of Medicine and Health Sciences, School of Nursing and Midwifery, Kigali, Rwanda

## Abstract

**Introduction:**

Worldwide, End Stage Renal Disease (ESRD) has become a public health concern increasing the number of patients maintained on hemodialysis prior to renal transplantation. Nonadherence to hemodialysis continues to impact on the care of ESRD patients, causing high increase in morbidity and mortality.

**Purpose of the Study:**

The purpose of this study was to determine the level of adherence to hemodialysis and the associated factors among End Stage Renal Disease (ESRD) patients in selected nephrology units in Rwanda.

**Methods:**

This was a descriptive cross-sectional design involving 41 participants. Participants were recruited using a purposive sampling technique. Demographic and adherence to hemodialysis data were collected with the use of structured interview schedules. Descriptive statistics were used to describe the demographic variables and the level of adherence to hemodialysis. Inferential statistics of chi-square was used to establish factors associated with adherence to hemodialysis.

**Results:**

Twenty-one (51%) of ESRD participants adhered highly (scores < 80%) to HD. Seventeen (42%) adhered moderately (70–79%) to HD while three (7%) had low level of adherence to HD (below 70%). The factors associated with adherence to hemodialysis were age (mean = 27; 95% CI 26.76–29, 17;* p* = 038) and religion (95% CI 26.29–60.12,* p* = 003). Frequencies of education of health care workers about the importance of not missing dialysis (95% CI 26.71–42.56,* p* = .000), perceived relative importance of hemodialysis (95% CI 20.44–27.76,* p* = .020), and experiencing difficulties during the procedure (95% CI 20.80–28.36,* p* = .004) were significantly associated with adherence to hemodialysis*. Conclusion.* Adherence to hemodialysis is still a public health concern in Rwanda. Health care providers and particularly nurses should continue to advocate for adherence to HD for better health outcomes. Further research is needed to identify the barriers to HD in Rwanda.

## 1. Introduction

End Stage Renal Disease (ESRD) is a known increasing public health concern globally [1]. The irreversible advanced CKD leads to End Stage Renal Disease (ESRD) where there is permanent loss of kidney function causing extreme mortality rates among this population [2]. The increasing prevalence of ESRD is similar to the increasing prevalence of type 2 diabetes mellitus which further complicates into ESRD as the total number of people with diabetes is expected to grow from 336 million in 2012 to 522 million in 2030 [3]. The increase of ESRD patients necessitates management on dialysis for better outcomes, thus making adherence to prescribed treatment essential [4]. Although kidney transplantation is the best choice of treatment of renal failure, resource constraints and shortage of kidney donations remain an issue [5]. Nevertheless, hemodialysis is also expensive but the preferred modality of treatment of ESRD patients in Rwanda [6].

In 2015, Rwanda Demographic Health Survey data showed a projected total population of 11,274,221 people with approximately 84 percent of them living in rural area. It is also evident that there is little or nothing known about the proportion of people living with ESRD or requiring RRT in Rwanda. From the national statistics, the majority of the people live in rural areas and yet the majority hemodialysis services for them are available in urban setting of Rwanda. There are four (4) dialysis units in Rwanda for which three are in the city center of Kigali and one in the rural setting in the southern province. There are approximately twenty working machines in the three dialysis units in the city center of Kigali and six (6) in the southern province [7]. This makes it difficult for far away rural populations in other provinces to access hemodialysis services, forcing the majority of the patients with ESRD to go to urban dialysis centers.

Nonadherence to hemodialysis on the other hand remains a major obstacle in the management of End Stage Renal Disease (ESRD) population. Documented literature reveals that approximately 50% of individuals with ESRD undergoing hemodialysis (HD) were not adhering to their prescribed treatment regimen [8]. This is also confirmed by Ibrahim and colleagues, who showed that nonadherence through skipping hemodialysis sessions ranged from 7 to 32% among ESRD patients [9]. Similarly, a study conducted on Zimbabweans showed that more than 50% of patients were not adhering to the scheduled hemodialysis plan. In fact, 93% of the respondents had missed at least one session of HD with 61% missing most of the scheduled sessions. Only seven percent had attended to all the hemodialysis sessions as scheduled. Sixty-seven percent had rescheduled the prescribed hemodialysis sessions more than once [10].

According to Duong et al. [11], nonadherence to treatment plan among patients with ESRD was problematic with approximately half of patients missing their sessions. Eleven percent (11 %) of the patients required extra treatment and 12 % had shortened their sessions. Negative patient outcomes and increased health care expenses as well as workload of the hemodialysis unit are consequences of nonadherence behaviors in ESRD population [12]. Numerous studies have also revealed that nonadherence is the cause of mortality, frequent hospitals visits, and hospital admissions [12, 13]. According to Abo et al. [4], missed and shortened dialysis treatment time resulted in physical problems such as hypotension, cramps, fatigue, and clots in access site.

Informal observations and clinical experience in Rwandan renal units reveal poor adherence to hemodialysis among ESRD patients. Moreover, there are limited studies in Rwanda about adherence to hemodialysis among ESRD. Yet, the health profile of Rwanda 2014 (WHO update) reveals that renal diseases were the fourteenth leading cause of death among 50 top causes of death in Rwanda [14]. Therefore, the aim of this study is to determine the level of adherence to hemodialysis and the associated factors among ESRD patients in selected hospitals of Rwanda.

## 2. Materials and Methods

### 2.1. Research Approach

This study used a quantitative approach to quantify the level of adherence to hemodialysis and the associated factors to adherence among ESRD population.

### 2.2. Study Design

This was a descriptive cross-sectional design in which the researcher collected and analysed quantitative data to determine the level of adherence to hemodialysis and associated factors among End Stage Renal Disease patients.

### 2.3. Study Sites

The study was conducted in three (3) selected referral dialysis centers in the city center of Kigali, Rwanda. The sites included one public hospital and two private settings, all of which are teaching, service, and research centers.

### 2.4. Study Population

Population is defined as all elements, such as individuals, events, or objects that meet the sample criteria for inclusion in a study, sometimes referred to as a target population [14]. In Rwanda, like in most of other African countries, there is limited data on the prevalence of ESRD requiring hemodialysis. However, from clinical observations, the number of ESRD patients on hemodialysis was approximately 70 nationwide at the time of the study. In this regard, all hemodialysis patients in Rwanda constituted the study population. The target population was patients attending hemodialysis at the selected study sites. The accessible population was patients attending hemodialysis at the time of the study.

### 2.5. Eligibility Criteria

The eligible respondents were those who were adult conscious patients who agreed to participate and had been on hemodialysis for more than 2 months as well as available at the time of the study. Respondents who were on Continuous Ambulatory Peritoneal Dialysis (CAPD), with Acute Kidney Injury (AKI) on hemodialysis, not in attendance at the time of the study, and critically ill and admitted were excluded from the study. There were some ESRD participants who were eligible but did not complete the interview schedule nor signed the informed consent form and thus were excluded from the study.

### 2.6. Sample Size

Quantitative researchers should select the largest sample possible so that it is representative of the target population. The number of patients on hemodialysis in Rwanda including those in private hospitals was 70 at the time of the study. The researchers only considered patients from the three referral hospitals who met the eligibility criteria and consented to participate in the study; hence a total sample size of 41 was used.

### 2.7. Sample Strategy

The researcher used purposive sampling to select a total population of study participants from dialysis units. This is whereby the entire population that meets the criteria is included in the research being conducted. The number of ESRD patients on hemodialysis were limited; hence the researcher used the total population. The researchers sampled all the hemodialysis patients from three selected units that met the inclusion and exclusion criteria.

### 2.8. Research Instrument

The research instrument for quantitative was developed using components of ERSD adherence questionnaire [15] and literature. The English instrument was translated into Kinyarwanda instrument by specialists in the Department of Languages at the University of Rwanda. Back translation into English version of the instrument was also conducted by an independent reader to ensure that there was no change in the meaning or misinterpretation caused by the translation. Self-reported method of collecting data was used. It was the structured interview guide that consisted of two sections, namely, demographics and level of adherence to hemodialysis. The demographic section captured the personal descriptive data of ESRD participations. The second section asked questions that revealed the extent of adherence to hemodialysis among ESRD patients.

Face validity was ensured through structuring the instrument into two separate stages. Content validity was ensured through giving the instrument to experts in the nephrology field to assess whether all contents to be measured have been included. Again, inclusion of items from literature also enhanced content validity of the instrument. Construct validity was achieved by checking items in the data collection tools against study objectives and concepts in the research instrument to ascertain whether all construct under study had been measured. Translating the research tool from English to local language ensured collection of reliable data, free from misinterpretation. Use of the structured interview schedule and following the items using the same wording and sequencing during the interview also enhanced the reliability of the data obtained through the instruments. A reliability analysis called Cronbach's alpha was performed to measure the internal consistency of the instrument. It was found to be 0.70 meaning that the instrument was a reliable measure of adherence to hemodialysis.

The instrument was designed to measure adherence to hemodialysis on a scoring system using a Likert scale. The minimum possible total score for adherence to hemodialysis was ten (10) and the maximum possible score, signifying perfect adherence to hemodialysis was thirty-four (34). Dividing the attained score on this section by the maximum possible attainable score (34) and multiplying by a hundred to come up with a percentage calculated adherence to hemodialysis. Adherence to hemodialysis of 90% to 100% was classified as high, 80% to 89% was classified as moderate, and adherence to hemodialysis below 80% was considered low. The researchers adopted the scale used by Chironda et al. [10].

### 2.9. Data Analysis

In this study, descriptive statistics were used to describe the extent of adherence to hemodialysis among ESRD patients. Inferential statistics of chi-square were used to test if there is any association between demographic variables and level of adherence to hemodialysis among End Stage Renal Disease patients.

### 2.10. Ethical Consideration

The permission was requested from ethical boards and research committee to carry out the study. Patient's rights were respected which include right to refuse or to withdraw from the study at any time without any consequences and they were prevented from discomfort and harm. Privacy and confidentiality were also observed. The purpose of the study was explained to the participants. Informed consent and participant's authorization were sought.

## 3. Results

### 3.1. Demographic Data

Table 1 shows the demographic characteristics of ESRD participants. Forty-one participants (Response Rate = 63%) with ESRD were selected and completed the study. Five (12%) were aged between 18 and 30 years, 9 (22%) were aged between 31 and 40 years, 6 (15%) were aged between 41 to 50 years, 11 (27%) were aged between 51 and 60 years, and 10 (24%) were aged greater than 60 years. The majority of the participants with ESRD were males [24 (59%)]. Regarding marital status, the majority, 28 (68%), were married. Four (10%) were not educated, 13 (32%) completed primary education, 16 (39%) were secondary educated, and 8 (20%) frequented colleges or universities. In terms of employment, 31 (76%) were unemployed, 6 (15%) were self-employed, and 4 (10%) were public servants. For 31 (76%) participants, the monthly income was less than 50000 Rwandan francs (< 58 USD), 3 (7 %) were having a monthly income between 50000 and 100000 Rwanda francs (approximately 58USD–116USD), 3 (7 %) had more than 100 000 and 200000 Rwanda francs (approximately 117USD–232USD) of monthly income, and 4 (10%) were having a monthly income of more than 200000 Rwanda francs (more than 232USD). Christians were representing the majority of the ESRD populations [39 (95%)] and Muslims were only 2 (5%). Eleven (27%) had ESRD for a period between three months and one year, 4 (10%) for one to two years, 6 (15%) for two to three years, 8 (20%) for three to five years, and 12 (29%) for more than five years.

### 3.2. Adherence to Hemodialysis among ESRD Participants

Regarding the number of dialysis sessions received per week in ESRD participants, 14 (34%) were receiving two dialysis sessions, 26 (64%) were receiving three sessions, and 1 (2%) was receiving four dialysis sessions per week (Table 2). According to the number of hours for each dialysis session, all 41 (100%) of ESRD participants remained on dialysis for 4 hours for each of the dialysis sessions (Table 2). With regard to the convenience of dialysis schedule for ESRD participants, 39 (95%) respondents agreed that the dialysis schedule was convenient for them while 2 (5%) participants expressed that the dialysis schedule was a burden to them (Table 2). With regard to the importance of not missing a hemodialysis session, 1 (2%) participant reported that he was never told the importance of not missing any dialysis session, 1 (2%) reported that he was told the importance of not missing a dialysis session for more than a month ago, 1 (2%) was told the importance of not missing a dialysis session for one month ago, 2 (5%) were told the importance of not missing a dialysis session for the past one week, and the majority [36 (88%)] were told the importance of not missing dialysis session during the week they were interviewed (Table 2).

About the importance of following a dialysis schedule, 1 (2%) participant reported that it was moderately important to follow dialysis schedule, 6 (15%) reported that it was very important, and 34 (84%) agreed that it was highly important to follow a dialysis schedule. Six (14%) ESRD participants reported having a lot of difficulty in staying for the entire dialysis session, 3 (7%) complained of having moderate difficulty, and 11 (27%) experienced little difficulty, while 21 (51%) reported having no difficulty in staying for the entire dialysis session. The difficulties experienced were mainly treatment related complications which include hypotension, muscle spasm, and pain at the insertion catheter site as well as headaches. On the number of dialysis sessions missed in the past month which was assessed using both self-report and hospital records, the study results showed that 2 (5%) ESRD participants missed 3 dialysis sessions, 5 (12%) missed 2 dialysis sessions, 9 (22%) missed one session, and 25 (61%) did not miss any dialysis session in the last month. Two (5%) ESRD participants shortened dialysis session once, while 39 (95%) did not shorten dialysis session in the last month.

### 3.3. Adherence Scores among ESRD Participants

Table 3 highlights the total adherence to hemodialysis scores of ESRD participants. The total adherence to hemodialysis score was 34 and the minimum expected adherence was 10 among ESRD participants. The maximum adherence to hemodialysis score obtained in the study sample was 29 out of 34, and the minimum adherence to hemodialysis score was 19 out of 34. The mean, median, and mode adherence to HD score were 26.65, 28, and 28, respectively. The researcher adopted an adherence scale to measure the level of adherence to HD among ESRD participants. Scale used was adopted from Chironda et al. [9], where 80 to 100% was identified as high adherence, 70 to 79% was identified as moderate adherence, and less than 70% was classified as low adherence. Based on the scale, 21 (51%) of ESRD participants scored above 80% meaning high adherence to hemodialysis. Seventeen (42%) scored between 70 and 79%, translating to moderate level of adherence to hemodialysis. Only 3 (7%) scored below 70% meaning that their level of adherence to HD score was low.

### 3.4. Factors Associated with Adherence to Hemodialysis in ESRD Population

Table 4 reveals the factors associated with adherence to hemodialysis in ESRD population. Results showed that age (*p* = .038) and religion (*p* = .003) of participants were statistically significantly associated with adherence to hemodialysis. Other demographic factors such as marital status (*p* = .971), educational level (*p* = .338), occupation (*p* = .375), and monthly income (*p* = .376) were not significantly associated with adherence to hemodialysis in ESRD population. In addition, frequencies of education by health care workers about importance of not missing dialysis (*p* = .000), perceived relative importance of hemodialysis (*p* = .020), and experiencing difficulties during the procedure (*p* = .004) were significantly associated with adherence to hemodialysis in the study.

## 4. Discussion

The findings from this study revealed low adherence in 49% of ESRD participants. The findings are consistent with findings from other studies that estimated 50% of patients on hemodialysis not adhering to at least part of their dialysis regimen [16, 17]. Similarly, thirty-nine percent of the study population missed their dialysis sessions at least once. This is also similar to the findings of Duong et al. [11] and Al-Khattabi who revealed 42% and 44% of ESRD patients that missed their dialysis sessions, respectively [18]. Contrarily, a study done by Tamie Nakao et al. [19] highlighted nonadherence rate of only 15 % among ESRD patients. It cannot be overstated that nonadherence has significant poor health outcomes and therefore patients with ESRD and undergoing hemodialysis should be encouraged to complete their dialysis sessions as prescribed. Findings of our study differ considerably from the findings of developed countries such as Japan and Sweden, where the missed dialysis sessions were nearly zero [17]. It is also noted that the shortening dialysis session in the present study was observed among 5% of the participants. This may be related to the technical problems faced by the dialysis machines since they need constant servicing.

Additionally, the findings of the study showed that age was statistically significantly associated with adherence to hemodialysis. However, it is noted that the effect of age is clinically quite small despite a statistically significant association that exists. The only difference seems to be in the mean ages between the age groups under 60s and over 60s. In this regard, participants of the ages of 41-50 years were observed to be the majority. The results are consistent with findings of Gerard et al. [6] and Chironda et al. [10], who revealed the average age of their patients as 45 and 46 years, respectively. The results are not surprising as it is important to note that individuals at this stage of life are beginning to make a significant impact of their lives; some of them have families and adherence is paramount to be able to support their families. Also, in developing countries, ESRD affects the population of under 50 years who are economically productive. Contrary to other studies, the mean age of patients with ESRD was 53 [20], whereas in the USA ESRD is more frequent in adult above 70 years, mainly due to longer survival rates among ESRD patients [21].

Again, the study results revealed that religion was significantly associated with adherence to hemodialysis. This is in line with a prospective study conducted by Freire de Medeiros et al. [22] that established religiosity to be associated with adherence to dialysis. The majority of ESRD participants were males rather than females. This is similar to the study findings by Chironda et al. [10] who revealed that the males were representing 57% and 43% were females. Contrary to these findings are the findings of Burkhalter et al. [23] that showed the predominance of females (65%). Yet for Duong et al. [11] study the males represented 47%. Gender was not associated with adherence to hemodialysis. However, this is in contrary to study done by Naalweh et al. [5] where male patients had significantly higher overall adherence scores than females (*p* = 0.034).

Varying levels of education were not significantly associated with the level of adherence to hemodialysis among ESRD population. This shows that ESRD affects both educated and noneducated people meaning that knowledge alone is not a predictor of adherence to hemodialysis [9, 19]. However, a decreased level of education can contribute to reduced levels of understanding leading to nonadherence and poor level of following medical instructions in favor of ESRD treatment [12]. On the contrary, increased level of education facilitates capturing and conveyance of information regarding concerns of the disease ESRD as well as importance of hemodialysis treatment.

Three-quarters of the participants were unemployed meaning that they did not have any monthly income. Moreover, there was no significant association between occupation, income, and adherence to hemodialysis among ESRD patients. However, dialysis in low income countries is an expensive procedure [24] and it is more likely that patients from low and middle income countries who cannot afford the dialysis sessions will have to skip some sessions of dialysis due to low economic status, considering, presently, that in Rwanda one session costs approximately over 100,000 Rwandan francs where only few Rwandans in need can afford hemodialysis treatment. This is the likely cause of nonadherence of hemodialysis among ESRD patients in Rwanda. Nevertheless, 49% of ESRD participants were covered by the fund for neediest survivors of genocide in Rwanda (FARG) which fully caters for all costs for hemodialysis without shortfall. However, 19% were self-sponsored, private medical insurances were covering 15%, and 10% of the participants were covered by the community based health insurance and these do not cater fully for hemodialysis treatment as patients are expected to pay the shortfall. Because of the high cost for hemodialysis treatment and lack of adequate health insurance [25], some patients ended up with missing or withdrawing from the treatment.

This situation is different from that of Europe, where the majority of ESRD patients on renal replacement therapy were covered at 100% [26]. In Georgia also RRT including hemodialysis therapy is covered by the state at 100% [27], as well as in US where ESRD patients are covered by Medicare without considering their age and in Libya and other developed countries where the access to dialysis therapy is free for ESRD Libyan patients [28]. The duration of ESRD was not associated with level of adherence to hemodialysis.

### 4.1. Limitations of the Study

Firstly, our study presents a smaller sample size that is related to the fact that patient number keeps dwindling depending on the financial capacity of the patients to maintain all hemodialysis sessions. Also, the number of patients that report at the hemodialysis centers is small, therefore, making data collection procedures quite challenging.

Our results also face a limitation of bias as we used face to face interview method for data collection. This might have introduced interviewer and information recall biases. Interviewer bias was mitigated by using three trained research assistants. Meanwhile, information bias was mitigated by the investigators sticking to the research instrument/protocol.

Thirdly, the fact that the study involved respondents on hemodialysis, asking them questions related to their adherence at the time of the interview may not necessarily mean they will adhere throughout the treatment regimen. Interviewers further tried to elicit questions to ascertain the willingness and ability of the patients to stay on hemodialysis.

## 5. Conclusion

Altered adherence to hemodialysis is still a big concern in Rwanda affecting negatively ESRD patients' treatment outcomes thus causing a huge burden on health care institutions. Age and religion were implicated to be significantly associated with adherence to hemodialysis. Health care providers and particularly nurses who care for patients and stay with them for longer hours need to advocate for patients with ESRD in view of completing their sessions for compliance and adherence to hemodialysis. Further research is required to identify barriers and promoters of adherence to HD among patients with ESRD in Rwanda.

## Figures and Tables

**Table 1:** Demographic characteristics of ESRD participants (N = 41).

Variable	Frequency (%)
**Age**	
18-30 years	5 (12%)
31-40 years	9 (22%)
41-50 years	6 (15%)
51-60 years	11 (27%)
Greater than 60 years	10 (24%)
**Gender**	
Male	24 (58%)
Female	17 (42%)
**Marital status**	
Married	28 (68%)
Single	7 (17%)
Separated	1 (2%)
Widowed	5 (12%)
**Level of education**	
Not educated	4 (10%)
Primary	13 (32%)
Secondary	16 (39%)
College/university	8 (20%)
**Occupation**	
Self-employed	6 (15%)
Public servant	4 (10%)
Unemployed	31 (75%)
**Monthly income (Rwandan Francs/USDs)**	
Less than 50000 (< 58USD)	31 (75%)
50000-100000 (58USD – 116 USD)	3 (7%)
More than 100000 to 200000 (>116USD – 232 USD)	3 (7%)
More than 200000 (> 232USD)	4 (10%)
**Religion**	
Christian	39 (95%)
Muslim	2 (5%)
**Duration of ESRD**	
3 months to 1 year	11 (26%)
More than a year to 2 years	4 (10%)
More than 2 years to 3 years	6 (15%)
More than 3 years to 5 years	8 (20%)
More than 5 years	12 (29%)
**Mode of payment for hemodialysis**	
Self-sponsored	8 (19%)
Government assisted	7 (7%)
FARG	20 (49%)
Private medical insurances	6 (15%)
Community based Health Insurance	4 (10%)

**Table 2:** Adherence to hemodialysis among ESRD participants (N = 41).

Variable	Frequency	Percentage (%)
**Days to receive dialysis**		
2 days or less	14	34
3 days	26	64
4 days	1	2
**Hours treated for each Session**		
4 hours	41	100
**Convenience of dialysis schedule**		
No	2	5
Yes	39	95
**Last day to be told the importance of not missing dialysis session**		
Never	1	2
More than a month ago	1	2
One month ago	1	2
Last week	2	5
This week	36	89
**Importance of following dialysis schedule**		
Moderate important	1	2
Very important	6	15
Highly important	34	83
**Difficulty of staying for the entire dialysis session**		
A lot of difficulty	6	15
Moderate difficulty	3	7
Little difficulty	11	27
No difficulty	21	51
**Missed Dialysis sessions during the last month**		
Missed three	2	5
Missed two	5	12
Missed one	9	22
None	25	61
**Shortened dialysis session during the last month**		
Once	2	5
None	39	95

**Table 3:** Adherence to hemodialysis scores among ESRD participants (N = 41).

**Adherence to HD out of 34**	**Adherence score percentage (%)**	**Level of adherence according to the scale**	**Frequency**	**Percentage frequency (%)**
**19**	56	Low	1	2
**22**	65	Low	1	2
**23**	68	Low	1	2
**24**	71	Moderate	3	7
**25**	74	Moderate	5	12
**26**	77	Moderate	6	15
**27**	79	Moderate	3	8
**28**	82	High	14	35
**29**	85	High	7	17
**Total**			41	100

**Table 4:** Associated factors of adherence to hemodialysis among ESRD participants.

**Associated factors**	**N**	**Mean**	**95% Confidence interval**	**P value**
**Age**				
18 -30 years	5	26.11	26.76 – 28.84	
41 – 50 years	9	26.17	25.06 – 27.16	.038^*∗*^
51 – 60 years	11	25.91	23.24 – 29.09	
Greater than 60 years	10	27.70	26.23 – 29.17	
**Religion**				
Christianity	39	26.90	26.29 – 27.50	.003^*∗*^
Muslim	2	22.00	16,12 – 60.12	
**Frequency of Education from health care workers for importance of not missing dialysis sessions**				
Every dialysis session	36	27.22	26.71 – 27.73	.000^*∗∗*^
Once a week	2	23.50	4.44 – 42.56	
**Relative importance of following sessions**				
Very Important	6	23.67	20.44 – 26.90	.020^*∗*^
Highly important	34	27.21	26.65 – 27.76	
**Experiencing difficulties during hemodialysis**				
A lot of difficulty	6	23.67	20.80 – 26.53	
Moderate difficulty	3	24.67	21.80 – 27.54	
Little difficulty	11	27.00	26.14 – 27.85	.004^*∗*^
No difficulty	21	27.62	26.88 – 28.36	

## Data Availability

The data used to support the findings of this study are available from the corresponding author upon request.
